# Manual therapists and people living with borderline personality disorder (BPD): a commentary on building safety, trust, and therapeutic effectiveness in physical care

**DOI:** 10.1186/s12998-026-00666-8

**Published:** 2026-07-27

**Authors:** Stanley Innes, Karthika Kasiviswanathan, Lee Daffin, Helen Mildred

**Affiliations:** 1 Adult Mental Health and Wellbeing Program, Eastern Health, Box Hill, VIC Australia; 2 Eastern Health Clinical School, Monash University, Box Hill, VIC Australia; 3 Spectrum Personality Disorder and Complex Trauma Service, Richmond, VIC Australia; 4 School of Allied Health, Murdoch University, Murdoch, WA Australia; 5 Deakin University, Burwood, VIC Australia; 6 Infant, Child and Youth Mental Health Service, Eastern Health, Box Hill, VIC Australia

**Keywords:** Borderline personality disorder, Musculoskeletal pain, Manual therapy, Professional-patient relations, Education, Professional

## Abstract

**Background:**

Borderline personality disorder (BPD) is a complex and often stigmatised mental health condition frequently comorbid with chronic pain and musculoskeletal disorders. Despite regularly encountering individuals with BPD, manual therapists (e.g., physiotherapists, chiropractors, osteopaths, and massage therapists) receive limited training on how emotional dysregulation, trauma, and relational sensitivity may influence physical-care encounters.

**Discussion:**

This clinically informed commentary explores how BPD’s psychological and neurobiological features intersect with bodily pain, therapeutic touch, and clinical communication. It highlights the potential for both therapeutic benefit and unintended harm within manual therapy interactions, and identifies a need for trauma-informed, psychologically attuned care. Drawing primarily on the National Institute for Health and Care Excellence (NICE) guideline for BPD (CG78, 2024 update), this paper cautiously adapts selected principles—such as collaboration, continuity, clear boundaries, and support for clinicians—to manual therapy contexts. The National Health and Medical Research Council (NHMRC) guideline (2012) is used selectively to provide additional operational detail where not explicitly addressed in NICE.

**Conclusion:**

While these considerations offer a pragmatic framework for enhancing safety and therapeutic engagement, their application requires careful adaptation to context, scope of practice, and resource availability. Further research, particularly co-designed with people with lived experience of BPD, is needed to evaluate feasibility and refine these approaches within manual therapy settings.

**Supplementary Information:**

The online version contains supplementary material available at 10.1186/s12998-026-00666-8.

## Introduction

Borderline personality disorder (BPD) is a complex and highly stigmatised condition with an estimated pooled global prevalence of 1.8% and is particularly over-represented in clinical populations [1, 2]. BPD is commonly conceptualised through both neurobiological and developmental frameworks. It is characterised by pervasive emotional dysregulation, an unstable sense of self, impulsivity, interpersonal difficulties, recurrent self-harm and suicidal behaviours, and a pronounced fear of abandonment [3, 4] (See appendix for full diagnostic criteria). Episodes of dissociation and transient stress-related paranoia are also common [3, 4]. According to Linehan’s biosocial theory, BPD develops from an interaction between a child’s biological vulnerability and an invalidating environment [5]. This interaction may contribute to intense emotions, difficulties with self-regulation, impaired self-concept and attachment security, and the development of unhelpful coping strategies over time [5].

This commentary reflects a multidisciplinary perspective that includes clinicians with extensive experience in manual therapy (SI, LD) and mental health care (SI, HM), academic expertise in personality disorders (HM), and lived experience expertise in BPD (KK). After an initial draft prepared by SI, all authors engaged in iterative revision and discussion, with lived experience expertise directly shaping the selection and adaptation of the NICE CG78 framework and the development of the clinical recommendations. Although the manuscript was not formally co-designed using a participatory research methodology, lived experience expertise was incorporated throughout the iterative development of the manuscript and contributed substantively to the conceptualisation and refinement of the recommendations presented.

Although manual therapy differs from psychotherapy in duration, structure, and scope, both involve repeated interpersonal encounters, embodied interaction, and the development of a therapeutic relationship. These shared relational and contextual features provide a rationale for cautiously and selectively adapting principles from mental health frameworks, while recognising important differences in training, expectations, and clinical boundaries. To our knowledge, no previous work has synthesised BPD clinical guidance specifically for manual therapy settings.

Manual therapists are defined here as health professionals who use hands-on techniques as a primary component of care, including physiotherapists, chiropractors, osteopaths, and massage therapists. Although these professions have evolved from distinct historical traditions and differ in training pathways, theoretical orientations, and regulatory frameworks across jurisdictions, they share several clinically relevant characteristics. These include the use of physical touch, close client proximity, and typically time-limited or episodic therapeutic relationships centred on musculoskeletal and related conditions. These shared features provide a rationale for considering manual therapy professions as a coherent group when examining relational, trauma-informed, and interpersonal aspects of care.

### Pain, the body, and BPD

People living with BPD frequently report chronic physical pain, including conditions such as back and neck pain, arthritis, and fibromyalgia [6]. One study found that nearly one-third of people presenting with chronic/persistent pain meet diagnostic criteria for BPD [7]. Although not required for diagnosis, a history of trauma, especially childhood maltreatment, is common among people with BPD and is strongly associated with chronic pain [8].

Neurobiological studies implicate dysregulation of limbic–prefrontal circuits responsible for emotion–pain modulation [9, 10]. This may be due to low natural resting levels of endogenous opioid activity in people with BPD, which is proposed to be associated with emotional dysregulation, subsequently contributing to inconsistent pain thresholds, sensory testing that is opposite to, or inconsistent with, what would normally be expected and an increased susceptibility to chronic pain [7, 10, 11]. Consequently, under emotionally distressing conditions, people with BPD may exhibit higher pain tolerance; yet during emotionally neutral conditions, pain perception can fluctuate markedly [12].

The bi-directional interactions between emotional distress, trauma history, and pain perception are shaped by dysregulated limbic–prefrontal pathways, altered internal body awareness, and stress-responsive neuroendocrine mechanisms [9]. As a result, people living with BPD who experience physical pain may require additional relational and clinical considerations to engage safely and consistently in treatments involving physical touch. These factors are particularly relevant in manual therapy, where care is often delivered through time-limited or episodic therapeutic relationships centred on bodily assessment and intervention. Clinicians must therefore remain attentive to both the emotional and physical dimensions of the treatment encounter [13].

However, navigating the intersection of BPD, trauma, and chronic pain may be challenging for practitioners with limited familiarity with personality disorders and the psychosocial factors influencing pain experience in this population. The extent of education and clinical exposure to BPD varies across manual therapy disciplines and jurisdictions, representing an important area for future research.

While these neurobiological and psychosocial models provide a useful framework for understanding the interaction between emotional dysregulation and pain in BPD, the evidence base remains evolving and heterogeneous. Much of the current literature is derived from theoretical models or studies with small and diverse samples, and findings should therefore be interpreted cautiously when applied across clinical populations and practice settings.

### Manual therapy, BPD and gaps in the literature

Despite the intersection of BPD, pain, and its biopsychosocial mechanisms, education on personality disorders remains largely absent from undergraduate manual therapy curricula [13–15]. Preliminary findings reported in a peer-reviewed conference abstract also suggest that trauma-informed and mental health-related educational content remains limited within North American chiropractic curricula, although these findings should be interpreted cautiously pending full publication [16]. Attitudinal research further underscores the importance of this educational gap. Health professionals, including those outside psychiatry, frequently hold more negative attitudes toward people with BPD than toward other diagnostic groups [17, 18]. As a result, many healthcare practitioners may work without a clear understanding of how the relational sensitivity and trauma histories associated with BPD can influence multiple components of care, including communication, consent, patient education, treatment selection, therapeutic engagement, and perceived treatment outcomes. In manual therapy settings, these factors may be especially relevant because care often involves physical touch, bodily exposure, close proximity, and repeated interpersonal interactions. Without adequate training and awareness, such dynamics may contribute to stigma, distress, therapeutic disengagement, and inconsistent care [17, 18].

Although the diagnostic label remains controversial, with debate about heterogeneity and stigma, the lived reality for many people with BPD involves recurrent crises and strained therapeutic relationships [19]. Stigma is pervasive: health professionals frequently report frustration, avoidance, or pessimism when engaging with this patient population, which in turn exacerbates shame and service disengagement [20]. Educational interventions have been shown to improve practitioners’ empathy and confidence, underscoring the importance of targeted training and reflective practice [20].

### Purpose

This commentary aims to raise awareness among manual therapists about the intersection of BPD and manual‑therapy practice, and to advocate for a shift in professional education toward trauma‑informed, psychologically attuned approaches. This shift is proposed in response to the current under-representation of trauma-informed and psychological content within many manual therapy curricula. Such a shift would better equip manual therapists to provide compassionate, collaborative care that upholds interpersonal boundaries and supports the unique needs of people living with BPD.

With the introduction establishing the context and outlining how emotion-regulation difficulties intersect with pain experience and body awareness in people with BPD, the subsequent sections build on this foundation by detailing the clinical features of BPD most likely to influence therapeutic engagement and by presenting practical strategies to help manual therapists enhance safety, empathy, and effectiveness in physical care.

## Engaging people with BPD and chronic pain in manual therapy

Understanding the psychological and relational dimensions of BPD is an essential first step for manual therapists working with people who have BPD and co‑occurring chronic physical pain [21]. Without this understanding, protective responses to perceived rejection or loss of control (e.g., missed appointments, heightened emotional intensity, sudden mistrust) may be misinterpreted by practitioners as clients’ therapy interfering behaviours, reinforcing stigma [22, 23]. Viewing these reactions as expressions of distress rather than defiance reframes the clinical encounter and promotes compassionate boundary-aware engagement. With this context, manual therapists may also be well placed to support re‑engagement in treatment when disengagement arises in the context of BPD.

Chronic hyperarousal, dissociation, and altered internal body awareness are often associated with BPD, and by distorting body perception, may intensify somatic symptoms [24]. These factors complicate assessment and can contribute to therapeutic frustration if clinicians interpret variable pain reports as malingering or exaggeration. From a trauma-informed standpoint, the body may serve as a site where distress is experienced, as well as the medium through which regulation is sought [25–27]. Since touch, movement, and somatic attention are core elements of manual therapy, they hold potential to support regulation and trust, but also to evoke vulnerability or re-traumatisation if not used sensitively [25].

While understanding the biopsychosocial mechanisms behind chronic pain in people with BPD provides a contextual basis for the client’s treatment journey, they offer limited practical direction for manual therapists. The National Institute for Health and Care Excellence (NICE) Guidelines, UK (2024 update) provide a current, comprehensive, internationally recognised evidence-based framework for working safely and compassionately with people living with BPD [28]. Its principles emphasise hope, collaboration, continuity, and supervision, which may offer useful considerations for communication, professional boundaries and therapeutic engagement within manual therapy settings. NICE CG78 was developed for specialist mental health services and assumes access to multidisciplinary teams, structured supervision, and continuity of care that may not be present in manual therapy settings, particularly in private practice. As such, its application requires careful adaptation to ensure alignment with the scope, resources, and clinical context of manual therapists. A summary table of NICE principles with suggested applied examples for manual therapists can be found in the Appendix.

While the NHMRC Clinical Practice Guideline for the Management of BPD (2012) remains influential in Australian clinical contexts, it has not been updated in recent years and no longer represents the Council’s current position [29]. In this manuscript, it is therefore used selectively to provide additional operational detail (e.g., care planning and risk management) where not explicitly addressed in NICE CG78. The intent is to integrate internationally recognised evidence with practice-relevant insights applicable to physical healthcare settings without extending the scope of manual therapists beyond their professional competencies.

## Key considerations

The following considerations serve as a starting point for how manual therapists might align with established mental-health standards. However, further empirical research, especially co-designed with people who have lived experience of BPD, is essential to validate and refine these considerations. Such participatory inquiry would ensure that future recommendations are not only theoretically sound but also grounded in the experiences, safety needs, and therapeutic preferences of those most affected.

Notably, these recommendations are not intended to extend manual therapists’ roles into psychological treatment, but rather to support safe, trauma-informed physical care within existing professional scope.

### Building and maintaining a therapeutic alliance

Key challenges experienced by tertiary mental health care providers, that will likely also be challenges for manual therapists include: a lack of understanding of the underlying drivers of distress, trouble differentiating acute from chronic risk, and having insufficient skills for de-escalating negative arousal and establishing positive engagement [17]. Consequently, it is unsurprising that developing an optimistic and trusting relationship is a central tenet of the NICE guideline for BPD [28]. People with BPD often have a history of invalidation, rejection, and trauma, and these histories can amplify their sensitivity to interpersonal dynamics [4, 5]. Manual therapy involves physical touch, proximity, and perceived authority, and these relational factors may evoke both hope and fear.

NICE recommends fostering treatment within an atmosphere of *hope and optimism*, emphasising that recovery is possible and attainable [28]. In the context of manual therapy, this begins with understanding BPD and its intersection with pain, followed by attentive responsiveness to the client’s needs, which may include emotional validation. Research from psychotherapy settings suggests that higher therapist responsiveness —that is, the therapist’s ability to be attentive to the patient, acknowledge and understand their concerns, respond to both the content and emotional tone of their communication, and interact in a caring, affirming, and respectful manner—is associated with stronger development of the therapeutic alliance in individuals with BPD [30]. While these findings are derived from structured mental health interventions and may not directly generalise to manual therapy contexts, they highlight the potential importance of attuned and responsive clinician behaviour in shaping engagement. In manual therapy settings, where training in managing complex emotional presentations may be limited, this underscores the need for heightened awareness of relational dynamics during early clinical encounters. This may not only help de-escalate their distress but also build a trusting working alliance.

People with BPD often struggle with fears of abandonment and find it difficult to trust others [4, 5]. Therefore, prioritising client psychological safety by maintaining consistent appointment times, using calm, transparent communication to facilitate collaborative care and validating both emotional and physical distress can allay many of the client’s concerns or fears.

A key feature of BPD is oscillating idealisation–devaluation patterns, where a client can make an initial strong bond with the health worker, then project anger, disappointment and distress if progress is not complete or fast enough, which may disrupt care [3]. Gentle but clear communication around interpersonal boundaries, especially with time, physical touch, and professional scope, will protect both client and practitioner. Shared decision-making is a key component of the NICE guidelines [28]. This can be operationalised by jointly setting treatment goals with the client, prioritising their agency throughout the treatment process (e.g., describing and gaining consent for touch, treatment intensity), and revisiting treatment goals regularly. Additionally, involving the client’s family or carer in the treatment process can support sustained engagement and functional outcomes where the client is not only adequately supported within the healthcare setting but also outside its confines [28, 31]. If appropriate, and with explicit client consent, inviting a support person to attend a session or review can enhance mutual understanding, treatment adherence, and sense of safety.

Taken together, an effective therapeutic alliance with people living with BPD can be built with consistency, clarity, and compassion—qualities widely recognised as crucial in both psychosocial and musculoskeletal care [32].

### Protecting practitioner and consumer wellbeing

Given the emotional intensity of therapeutic work, NICE guidelines recommend access to adequate training, supervision, and support for clinicians working with BPD [28]. People living with BPD may, at times, struggle with intense emotions in ways that can place additional demands on the therapeutic relationship [33]. These responses often reflect underlying distress rather than deliberate behaviour, and they can understandably challenge clinicians’ emotional boundaries, especially if they lack specialised training in working with complex emotional presentations.

Manual therapists, particularly those in solo or private practice, who often lack access to the structured supervision common in mental-health settings, may also experience emotional challenges when working with people living with BPD. Evidence suggests that many mental health professionals, including clinical psychologists and psychiatrists within Australia, report insufficient training to work confidently with people with BPD [34]. If these challenges exist even within mental-health professions, they are likely to be even more pronounced among manual therapists, whose training may include less exposure to complex emotional presentations and relational dynamics associated with BPD. As a result, practitioners may understandably feel under-equipped to recognise or respond to emotional distress that arises during treatment sessions. This gap reflects a broader educational and systemic limitation rather than individual inadequacy, and it may hinder clinicians’ ability to respond calmly and consistently when heightened emotional states occur in the context of care. Reflective practices (e.g., peer discussion, clinical supervision, journaling) may help practitioners process emotional responses and reduce the risk of compassion fatigue [35]. In addition to supporting practitioner wellbeing, reflective approaches may also enhance the clinicians’ ability to communicate in a calm, validating, and emotionally attuned manner during challenging clinical encounters.

In manual therapy contexts, reflective listening may involve simple but intentional communication strategies such as summarising the patient’s concerns, validating emotional and physical experiences, and checking understanding without attempting to provide psychological intervention, communication approaches that have been described across counselling and motivational interviewing literature [36, 37]. Given that many manual therapists work in solo or small group practices without access to formal supervision, reflective practice may be supported through peer discussion, case-based reflection, or continuing professional development activities. Where available, cross-disciplinary consultation or mentorship with mental health professionals (e.g., psychologists or psychiatrists) may provide additional support in managing complex emotional presentations, while remaining within professional scope. These strategies align with NICE recommendations, emphasising the value of routine supervision and staff support [28].

Professional boundaries are integral to both safety and ethical care. NICE advises clear care planning and defined roles among professionals (1.3.2) [28]. For manual therapists, this includes explaining the scope of physical treatment, limits of confidentiality, and pathways for escalation if emotional or behavioural risk emerges. Written documentation of informed consent, session content, and referrals is a standard expectation across most regulated manual therapy professions and jurisdictions, forming part of routine clinical governance and medico-legal practice. In addition to meeting professional and regulatory requirements, such documentation may provide structure and containment within the therapeutic encounter, particularly when working with individuals experiencing heightened emotional distress.

NICE also stresses the importance of differentiating between long-term and immediate risk (acute vs. chronic). An acute setting will be accompanied by signs of escalating distress (e.g., expressions of hopelessness, self-blame, or suicidal ideation). Neither the NHMRC nor NICE guidelines expect manual therapists to conduct formal suicide-risk stratification; instead, they should calmly express concern and compassion. The NHMRC guidelines provide additional operational detail regarding the use of collaboratively developed management or crisis plans in situations of acute risk [29]. This plan would contain information focused on strategies effectively used in the past to alleviate distress that bring about de-escalation so that the consumer does not leave the setting in distress. It helps the manual therapist to communicate with the client about what has helped before, encourages practical actions, offers follow-up for the pain-related symptoms, and escalates to mental health services if distress/risk is increasing. The contents of a typical Management Plan are outlined in the Appendix.

The NICE guidelines are more generic and recommend ensuring the person’s immediate safety, and liaising with consent with their general practitioner or mental-health service by having accessible local crisis contact details within the clinic .

While NICE and NHMRC guidelines do not typically position manual therapists as responsible for formal suicide-risk stratification, expectations may vary across jurisdictions and practice settings. In many manual therapy settings, a pre-existing crisis or management plan may not be available or accessible. In such situations, therapists should prioritise immediate safety and adopt a calm, supportive approach. This may include pausing treatment, acknowledging the person’s distress, and using simple grounding or reassurance strategies where appropriate. Therapists should then follow local protocols for escalation, which may include contacting the patient’s general practitioner, referring to emergency or crisis services, or seeking immediate assistance if there are safety concerns, in accordance with jurisdictional scope of practice and organisational requirements.

The NICE guidelines caution that transitions within, discharge out of and withdrawal of care can activate intense emotions for people with BPD who often struggle with fears of abandonment. Continuity and closure are particularly important. Manual therapists can prepare for this by confirming the total expected number of sessions early. Ending treatment should be structured, discussed early (with reminders), contain summaries (achievements, self-management, updating information with other practitioners) and clear guidance on how to access other supports and/or re-engage later. This offers a soft transition, as the client would have had time to mentally prepare themselves, reducing perceived abandonment and fostering continuity of care across the broader health system.

### Conducting care: practical considerations

NICE emphasises the principles of comprehensive assessment, collaborative planning, and calm crisis management, which are easily adapted to physical care environments.

Manual therapists should recognise that a formal diagnosis of BPD may not be known or disclosed in physical-care settings due to stigma or privacy concerns, and care should therefore be guided by observed needs and distress rather than diagnostic labels alone.

Initial assessment should include not only biomechanical findings but also awareness of psychosocial factors that may influence pain or treatment engagement. Simple open-ended questions (“How has stress been affecting your body lately?”) can elicit relevant information without delving into psychotherapy. NICE advises offering *post-assessment support* if sensitive topics arise. In manual therapy settings, this may involve allowing additional time at the end of a session, providing brief grounding or reassurance, and ensuring the patient leaves the consultation in a stable state. This does not extend to psychological intervention but reflects a supportive and ethically responsive approach within professional scope.

During treatment, therapists should explain what will happen before any physical contact, checking consent frequently, and remaining alert to signs of dissociation or discomfort. NICE’s recommendation to *maintain a calm and non-threatening attitude* during crises applies equally if distress escalates. It is prudent to pause treatment, validate the emotion, and collaboratively decide whether to continue, stop, or modify care. Avoiding minimisation (“You’ll be fine”) and providing empathy (“I can see this feels overwhelming”) are small but powerful acts that model support and shared control.

Inter-professional collaboration is also consistent with NICE. Where chronic pain, trauma, or comorbidity is evident, therapists should liaise (with consent) with the person’s medical practitioner, psychologist, or psychiatrist to support coordinated care, consistent with broader recommendations for interprofessional collaboration in healthcare. Such collaboration may reduce fragmentation of care and support integration of physical and mental health management.

Finally, avoiding re-traumatisation requires sensitivity to power dynamics. Respecting autonomy, maintaining choice, and explaining each step of care uphold the NICE principles of transparency and informed participation.

### Implications for manual therapy education and practice

NICE explicitly calls for specialist teams to provide training programs covering diagnosis, management, and stigma reduction for all professionals who encounter people with BPD. The absence of equivalent content in many manual-therapy curricula represents an important yet closable gap. While approaches such as simulation, reflective practice, and interdisciplinary learning are well established within broader health professional education, there is currently limited direct evidence regarding their impact within manual therapy training contexts. As such, the suggestions outlined here should be considered as pragmatic, evidence-informed starting points rather than prescriptive solutions.

## Undergraduate and entry-level education

Undergraduate and postgraduate programs could enact learning outcomes on:

- Recognition of emotional dysregulation and trauma-related behaviours,
- Principles of therapeutic boundaries and risk awareness,
- Communication strategies grounded in validation and collaboration,
- And referral pathways within local mental-health systems.

Simulation, reflective writing, case-based learning, and interdisciplinary placements could cultivate the psychological literacy needed for trauma-informed manual therapy. For example, undergraduate and entry-level programs could incorporate simulated case management scenarios using trained actors or people with lived experience portraying individuals with BPD and chronic pain. These scenarios could allow students to practise communication skills such as validation, boundary setting, collaborative decision-making, and responding to emotional distress within a safe learning environment. Similarly, case-based discussions involving complex presentations could help students develop clinical reasoning around trauma-informed care, interpersonal dynamics, and appropriate referral pathways.

## Continuing professional development and early career support

In addition to undergraduate training, there is a need to support practising manual therapists through continuing professional development opportunities that address trauma-informed care, communication strategies, and management of complex presentations. Early career clinicians may particularly benefit from observing and modelling approaches used by experienced practitioners.

In Australia, existing continuing professional development (CPD) frameworks across chiropractic, physiotherapy, and osteopathy already recognise activities such as workshops, reflective practice, peer discussion, case reviews, and interdisciplinary education. These established structures may provide feasible mechanisms for integrating educational initiatives related to trauma-informed care and working with people living with BPD. For example, simulation-based workshops using trained actors or consumer educators portraying individuals with complex pain and emotional presentations could allow practitioners to practise validation, boundary setting, and collaborative communication. Similarly, case-based discussion groups and reflective writing activities could facilitate critical reflection on therapeutic relationships, stigma, and management of emotionally challenging encounters. Interdisciplinary training delivered alongside psychologists, psychiatrists, or lived experience practitioners may further strengthen clinicians’ confidence and understanding of complex emotional presentations.

However, to our knowledge, there are currently few, if any, CPD initiatives specifically designed to prepare manual therapists for working with people living with BPD. The development and evaluation of such educational programs therefore represents an important future direction for manual therapy education and professional development.

Simulation, reflective writing, and interdisciplinary placements could cultivate the psychological literacy needed for trauma-informed manual therapy. Professional associations and accrediting bodies could also issue practice guidance consistent with NICE CG78, outlining expectations for working safely with people who have complex emotional needs. Recommendations for professional guidance aligned with NICE CG78 should therefore be interpreted in the context of jurisdictional scope of practice, regulatory standards, and local healthcare systems, which may differ substantially across countries and professions.

## Co-training and interdisciplinary collaboration

Partnerships with mental health professionals, including psychologists and psychiatrists, may support cross-disciplinary learning, supervision, and co-designed training initiatives. Leveraging existing educational resources developed for behavioural health professionals may provide a pragmatic and scalable approach to enhancing competencies within manual therapy.

From a systems perspective, NICE advocates involving people with lived experience and their families in training and service planning. Manual-therapy education could adopt a participatory approach by inviting consumer educators to share experiences of receiving physical care during emotional distress, helping students understand the relational nuances of touch, trust, and safety.

Embedding NICE principles within manual-therapy education will cultivate practitioners who are not only biomechanically proficient but also psychologically aware and ethically grounded. Acquisition of these skills could better align manual therapists as integral contributors to multidisciplinary, recovery-oriented care for people living with BPD. Table 1 summarises some practical strategies for manual therapists to consider adopting from the NICE guidelines.

**Table 1:** Key practical takeaways for manual therapists working with those with BPD

NICE principle	Practical example (Manual-therapy context)
Establish consistency & predictability	May involve maintaining consistent appointment times, clearly explaining treatment plans, describing and gaining consent for each intervention, and providing advance notice of transitions within or out of care to support safety and trust.
Validate experiences	May include acknowledging both physical pain and emotional distress without judgement, using empathic and non-minimising language to reduce shame and support engagement.
Set clear and supportive boundaries	May involve explaining the scope of treatment, session duration, and professional limits early, and communicating these boundaries in a calm and transparent manner.
Support clinician reflection and wellbeing	May include engaging in peer discussion, case reflection, or supervision (where available) to process challenging encounters and reduce the risk of emotional fatigue.
Collaborate and coordinate care	May involve liaising (with consent) with general practitioners, psychologists, or psychiatrists to support coordinated care and communication across providers.
Ensure consent and sensitivity in physical care	May include explaining the nature, location, and purpose of physical contact before treatment, checking consent regularly, and remaining attentive to signs of discomfort or distress.
Provide supportive responses to distress	If distress arises, this may involve pausing treatment, acknowledging the person’s experience, using simple grounding or reassurance strategies, and ensuring the patient leaves the session in a stable state.
Respond to risk within scope	In the absence of a known crisis plan, therapists may prioritise immediate safety and follow local escalation pathways (e.g., contacting a general practitioner or emergency services), in line with jurisdictional scope and organisational requirements. Where available, collaboratively developed management or crisis plans may help guide responses to distress or risk.

*Abbreviations* BPD, Border line personality disorder

This commentary synthesises existing guideline recommendations and clinical insights rather than presenting new empirical data. The applicability of these considerations may vary across professional contexts, regulatory frameworks, and individual client circumstances. Empirical research is required to evaluate their feasibility and effectiveness in manual-therapy settings.

## Conclusion

This commentary highlights the potential value, and necessary caution, of adapting established mental health principles to manual therapy contexts. While frameworks such as NICE CG78 provide a useful foundation, their application requires careful consideration of differences in clinical setting, scope of practice, and resource availability, particularly in private practice environments.

Adapting selected NICE principles to manual therapy settings may offer a practical pathway to enhance safety, therapeutic engagement, and emotionally attuned care. Establishing clear boundaries, maintaining transparent communication, validating distress, and ensuring access to supervision or reflective support may benefit both practitioners and consumers. However, these considerations represent only an initial translation of mental-health principles into physical-care practice.

Future progress must move beyond adaptation toward co-creation. Research co-designed with people who have lived experience of BPD is essential to refine these considerations, evaluate their feasibility in real-world clinical environments, and articulate best-practice approaches specific to manual therapy contexts. Such collaboration will help ensure that care remains evidence-informed, person-centred, safe, and restorative for both physical and emotional wellbeing.

## Supplementary Information

Below is the link to the electronic supplementary material.

Supplementary Material 1

## Data Availability

No datasets were generated or analysed during the current study.
