# Supplementary material for: Manual therapists and people living with borderline personality disorder (BPD): a commentary on building safety, trust, and therapeutic effectiveness in physical care
Source: Chiropr Man Therap. 2026 Jul 27;34:27. doi: 10.1186/s12998-026-00666-8 (PMC13412130; doi:10.1186/s12998-026-00666-8)
Supplement: Supplementary file 1 — Supplementary Material 1 [file 12998_2026_666_MOESM1_ESM.docx]

**Appendix Table: Relevant NICE Guideline principles with suggested applied examples for manual therapists**

| **NICE Principle (CG78 section)** | **Suggested applied example for Manual Therapists** |
| --- | --- |
| **1.1.3 Develop an optimistic and trusting relationship** | May involve maintaining consistent scheduling, using a calm tone, communicating transparently, and expressing realistic optimism about recovery. This may also include acknowledging both emotional and physical aspects of pain to support engagement and trust. |
| **1.1.4 Involving families or carers** | Where appropriate and with consent, may involve including family members or support persons to assist with understanding care plans, supporting attendance, or facilitating continuity of care. |
| **1.1.5 Post-assessment support** | Where sensitive or distressing topics arise (e.g., discomfort with touch), may involve allowing time for the person to settle before the session ends, and offering brief grounding or reassurance where appropriate to ensure the patient leaves in a stable state. |
| **1.1.6 Managing endings and transitions** | May involve discussing the expected number of sessions early, providing advance notice of transitions, and offering summaries, referral options, and guidance on re-engagement to reduce distress associated with endings. |
| **1.1.8 Training, supervision and support** | May include engaging in peer discussion, case reflection, or supervision (where available), as well as participating in continuing education related to trauma-informed care and working with complex emotional presentations. |
| **1.3.1 Assessment** – Holistic psychosocial evaluation. | May include awareness of psychosocial factors (e.g., stress, sleep, mood) that may influence physical symptoms, using simple, non-intrusive questions where appropriate. |
| **1.3.2 Care planning** – Collaborative, realistic goals. | May involve co-developing treatment goals that prioritise function and self-management, while clearly defining treatment scope, boundaries, and escalation pathways where relevant |
| **1.3.3 Responding to distress and potential risk** | May involve recognising signs of distress (e.g., dissociation or emotional escalation), maintaining a calm and supportive approach, and ensuring appropriate referral pathways are accessible. Documentation of concerns should occur in line with professional and regulatory requirements. |
| **1.3.4 Therapeutic structure and supervision** | May involve maintaining a clear and predictable session structure, explaining each intervention before implementation, checking consent regularly, and seeking peer support or supervision to reflect on challenging encounters. |
| **1.3.6 Comorbidity management** | Where appropriate and with consent, may involve liaising with general practitioners or mental health providers to support coordinated care where physical symptoms intersect with mental health conditions. |
| **1.3.7 Crisis management** | Where distress escalates, this may involve pausing treatment, maintaining a calm and supportive stance, validating the person’s experience, and drawing on previously discussed strategies where available. Where concerns about safety persist, therapists may follow local escalation pathways (e.g., contacting a general practitioner or emergency services), in line with scope of practice. |
| **1.3.9 Discharge to primary care** | May involve providing written advice on self-management strategies, outlining pathways for re-engagement, and supporting continuity of care beyond the manual therapy setting. |

**Abbreviations:** NICE - National Institute for Health and Care Excellence guideline for BPD (CG78, 2024 update)

**Appendix: Management Plan Content** (Adapted from The National Health and Medical Research Council (NHMRC) Guideline, 2012)

Where a collaboratively developed management or crisis plan is available, it may include the following elements (adapted from the National Health and Medical Research Council guideline, 2012, section 8.4):

- Diagnosis and any co-existing mental health conditions
- Short-term and long-term treatment goals
- Situations that may trigger distress or increase risk
- Self-management strategies that may reduce distress or risk
- Strategies that have previously been helpful or unhelpful
- Emergency contacts and crisis support options
- Health professionals, services, and agencies involved in care
- Roles of family members, carers, or other supports (where relevant)
- Planned review timeframes
- Identification of who holds a copy of the plan

**Footnote:** These examples represent practice-informed adaptations of NICE CG78 principles and are not intended to extend the scope of manual therapy into psychological treatment. Application will vary depending on jurisdiction, training, and clinical context.

**Possible “pop-up Box 1” Diagnostic Features of Borderline Personality Disorder (DSM-5)**

**Borderline Personality Disorder (BPD)** is defined in the *Diagnostic and Statistical Manual of Mental Disorders (DSM-5)* as a pervasive pattern of instability in interpersonal relationships, self-image, and affect, along with marked impulsivity, beginning by early adulthood and present across contexts.

A diagnosis requires five (or more) of the following:

1. Frantic efforts to avoid real or imagined abandonment
2. Unstable and intense interpersonal relationships, characterised by alternating between idealisation and devaluation
3. Identity disturbance, including unstable self-image or sense of self
4. Impulsivity in at least two potentially self-damaging areas (e.g., spending, substance use, reckless behaviour)
5. Recurrent suicidal behaviour, gestures, threats, or self-harming behaviour
6. Affective instability, due to marked reactivity of mood (e.g., intense episodic dysphoria, irritability, or anxiety)
7. Chronic feelings of emptiness
8. Inappropriate, intense anger or difficulty controlling anger
9. Transient, stress-related paranoid ideation or severe dissociative symptoms

**Clinical note:** These features may present variably across individuals and contexts. In manual therapy settings, aspects such as emotional reactivity, interpersonal sensitivity, dissociation, and fear of abandonment may be particularly relevant to therapeutic engagement, communication, and responses to physical touch.
